# Strategies for complete plastid genome sequencing

**DOI:** 10.1111/1755-0998.12626

**Published:** 2016-11-28

**Authors:** Alex D. Twyford, Rob W. Ness

**Affiliations:** ^1^ Institute of Evolutionary Biology Ashworth Laboratories University of Edinburgh Edinburgh EH9 3FL UK; ^2^ Department of Biology University of Toronto Mississauga Mississauga ON Canada

**Keywords:** chloroplast, genomic, next‐generation sequencing, plant evolution, plastid

## Abstract

Plastid sequencing is an essential tool in the study of plant evolution. This high‐copy organelle is one of the most technically accessible regions of the genome, and its sequence conservation makes it a valuable region for comparative genome evolution, phylogenetic analysis and population studies. Here, we discuss recent innovations and approaches for de novo plastid assembly that harness genomic tools. We focus on technical developments including low‐cost sequence library preparation approaches for genome skimming, enrichment via hybrid baits and methylation‐sensitive capture, sequence platforms with higher read outputs and longer read lengths, and automated tools for assembly. These developments allow for a much more streamlined assembly than via conventional short‐range PCR. Although newer methods make complete plastid sequencing possible for any land plant or green alga, there are still challenges for producing finished plastomes particularly from herbarium material or from structurally divergent plastids such as those of parasitic plants.

## Introduction

DNA sequences of plastids have provided many important insights into plant ecology and evolution over the past three decades (Palmer 1987; Chase *et al*. 1993; Petit & Vendramin 2007; Hollingsworth *et al*. 2016). The continued popularity and utility of plastid sequencing is due to properties that make it the most accessible genome to the plant molecular biologist. The highly conserved gene order, near absence of recombination and low levels of nucleotide substitution (Box 1), make the plastid the ideal target for universal primers that amplify homologous loci in phylogenetically divergent species (Palmer 1987; Taberlet *et al*. 1991; Clegg *et al*. 1994; Shaw *et al*. 2005). In addition, the high‐copy number of plastids per cell means that genomic DNA extracts are naturally enriched for plastids (Bendich 1987) and thus an easier target than low‐copy nuclear genes for sequencing, particularly from small or degraded samples (Staats *et al*. 2013). Although attention is shifting from the sole‐reliance on plastid genes, to exploiting DNA variation in the nuclear genome (Hollingsworth *et al*. 2011; Lemmon & Lemmon 2013; Mandel *et al*. 2014; Weitemier *et al*. 2014), many research fields such as phylogenetics and phylogeography will continue to use plastid sequences for both technical and biological reasons.

Box 1 **Typical and atypical plastid genome structures**

Land plant plastomes are typically considered to be 120–160 Kb in the length, nonrecombinant, circular, maternally inherited, strongly AT‐biased and with highly conserved gene order. While these general observations hold for many species, there are notable exceptions to each of these generalities, for example presence of recombination (Maréchal & Brisson 2010; Ness *et al*. 2016), noncircular plastids (Lilly *et al*. 2001), biparental plastid inheritance (Metzlaff *et al*. 1981), giant plastomes (e.g. chlorophyte green alga *Floydiella terrestris*, 521 Kb plastome sequence, Brouard *et al*. 2010) and miniaturized plastids <100 Kb (Wicke *et al*. 2013).

Most plastids are organized into a long single copy section (LSC) and a short single copy section (SSC), typically flanked by two inverted repeats (IRs) ~20–25 Kb long (Kolodner & Tewari 1979). These IRs are the most prominent structural feature of the plastome and appear to be maintained by concerted evolution and thus are near identical in their sequences. However, it is important to note that some groups have lost part of one, all of one or both inverted repeats (Palmer *et al*. 1987).

Plastomes are generally repeat poor and do not contain long repeats outside of the IR. For example, *Camellia* plastids contain just 156 repeats over 30 bp in length, with the longest repeat 82 bp long (Huang *et al*. 2014). Seldom are repeats longer than current sequence read length, with rare exceptions (e.g. longest repeat in *Hordeum vulgare* is 540 bp, Saski *et al*. 2007). Short repeats are also present and may be used as a variable marker in population studies. A/T mononucleotide repeats are the most abundant form of repeat, with 700 such repeats over 8 units in length in the alga *Chlorella vulgaris* (Wheeler et al. 2014).

Many biological properties of the plastid make them ideal for ecological and evolutionary studies. For example, predominantly uniparental inheritance makes plastid sequences informative for population genetic studies investigating seed flow (Ennos 1994; Petit *et al*. 2005), and low effective population sizes and thus short coalescent times make it ideal for phylogeography (Petit & Vendramin 2007). More generally, the plastid contains a core set of genes for photosynthesis, protein synthesis and ribosome production, and thus, plastid studies provide insights into key biochemical pathways and cellular functions (Kleffmann *et al*. 2004; Naumann *et al*. 2016). Plastid sequencing can also reveal the cyanobacterial origins of plastids and the genomic changes associated with endosymbiosis (McFadden 2001). As such, sequencing plastid loci has been instrumental in improving our understanding of phylogenetic relationships (Palmer 1987; Chase *et al*. 1993; Jansen *et al*. 2007), phylogeographic patterns (Soltis *et al*. 1997; Petit & Vendramin 2007), species discrimination (Hollingsworth *et al*. 2009; Nock *et al*. 2011), hybridization (Palme *et al*. 2004), photosynthesis (Leister 2003) and genome evolution (Wicke *et al*. 2011, 2016).

In each of these research fields, the move from the analysis of single‐gene regions that can be amplified by PCR, to complete plastid genomes (plastomes), is important to provide higher resolution and address previously unanswered questions (Hollingsworth *et al*. 2016). For example, recent studies have shown that (near) complete plastid DNA sequences improve phylogenetic support in analyses of recent rapid radiations (Parks *et al*. 2009; Barrett *et al*. 2014) and increase the ability to discriminate species with DNA barcoding (Ruhsam *et al*. 2015). Complete plastid sequences facilitate the study of mechanisms of gene loss and genome evolution in lineages where the plastid is subject to an altered selection regime, such as parasitic, carnivorous and mycoheterotrophic plants (Box 1, Barrett & Davis 2012; Wicke *et al*. 2013, 2014). Complete plastid genomes are necessary for detecting intracellular gene transfer between plastids, mitochondria and the nucleus (Iorizzo *et al*. 2012; Straub *et al*. 2013; Ma *et al*. 2015; : Wysocki *et al*. 2015). Sequencing all plastid genes also allows the discovery of the most variable loci for phylogenetic and population genetic inference (e.g. plastid microsatellites, Provan *et al*. 2001), for use over different spatial and temporal scales (Parks *et al*. 2009; Doorduin *et al*. 2011; Zhang *et al*. 2011). Overall, the widespread interest in plastid genome sequencing, in conjunction with improved sequencing techniques (discussed below), has led to a surge of published plastomes, with over 1000 available in GenBank, representing the full taxonomic scope of green plants and a more sparse sampling of other plastid bearing lineages (Donaher *et al*. 2009; Janouškovec *et al*. 2015; Smith & Keeling 2015).

**Figure 1:**
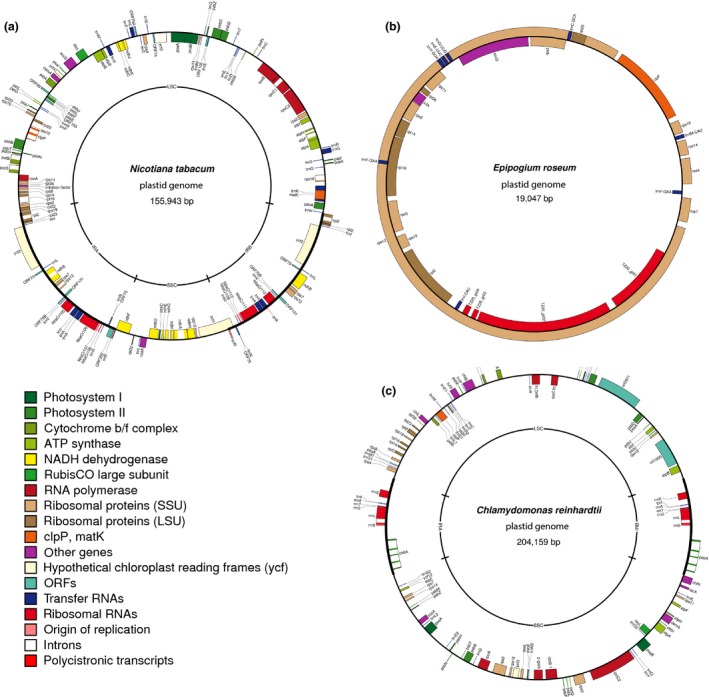
Structural diversity in plastids. (a) *Nicotiana* has a typical land plant plastid of 156 Kb and tripartite structure, (b) the mycoheterotrophic nonphotosynthetic orchid *Epipogium roseum* has the smallest plastid genome to date at 19 Kb, and with greatly reduced gene content, (c) green algae, here represented by *Chlamydomonas reinhardtii*, demonstrate dramatic plastid genome variation and include species with giant plastomes over 500 Kb in length. [Colour figure can be viewed at http://wileyonlinelibrary.com]

Plastid genome sequencing, like many areas of molecular biology, has been influenced by numerous technical innovations in DNA sequencing. The first complete plastid sequence was produced by sequencing overlapping clones from restriction endonuclease fragments of *Nicotiana tabacum* (Shinozaki *et al*. 1986; Fig. 1a). This approach was superseded by PCR amplification and Sanger Sequencing (Taberlet *et al*. 1991). Now next‐generation sequencing of total genomic DNA is emerging as a direct and cost‐effective way to assemble the complete plastid sequence for any plant species (Nock *et al*. 2011). However, the rapid development of many sequencing and bioinformatic approaches to recover the plastome sequence can lead to some confusion in choosing the most effective option. Here, we give examples and explain the underlying principles of the most popular approaches and provide recommendations for how to sequence the plastome of nonmodel species with minimal cost and effort. In particular, we consider strategies for when only a single plastid sequence is required, through to scalable approaches for retrieving plastid sequences for many individuals and species. With each of these approaches, we consider the end‐goal to be a complete plastid sequence free of sequencing gaps and errors. We start by outlining the potential approaches to retrieve the plastome (via enrichment and nonenriched samples, Box 2), before considering the suitability of different sequencing technologies and assembly approaches.

Box 2 **Selecting an enrichment approach**

A key decision for plastid assembly is whether to enrich samples for plastids through organelle isolation, PCR, hybrid baits or methylation enrichment, or to proceed with direct sequencing of a nonenriched genomic DNA extracts. Plastid enrichment introduces a time‐consuming (and potentially expensive) laboratory procedure, but has the benefit of focusing the downstream sequencing on the desired genomic regions, aiding analysis and reducing sequencing costs. In contrast, sequencing a total gDNA extract and subsequently identifying and assembling plastid reads may be faster and more cost‐effective in terms of laboratory procedures. However, it can be wasteful in terms of unnecessarily sequencing other genomic regions, reducing the multiplexing potential and also introduces an additional set of bioinformatic challenges. Typically, the choice of whether or not to enrich for plastids depends on a mixture of biological and technical aspects.

**Figure 2:**
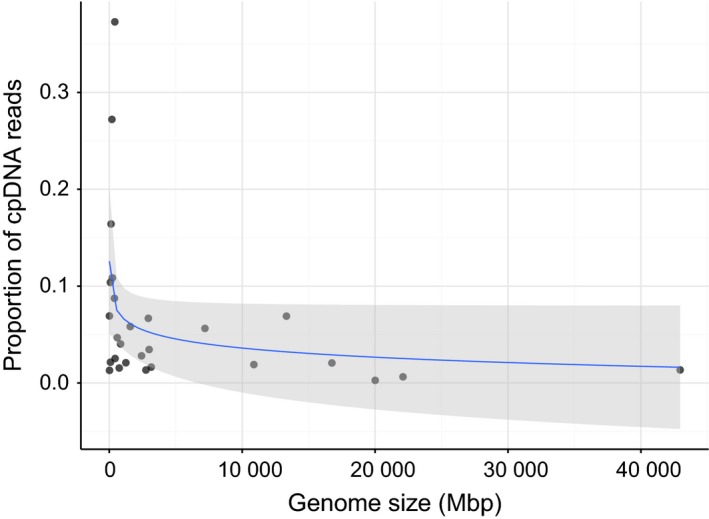
Representation of plastid reads in gDNA sequence libraries of species with different genome sizes. The graph shows the proportion of sequence reads from a phylogenetically diverse range of 27 green plant species that map to a reference database of 100 plant plastomes. Grey shading indicates the 95% confidence interval of the fitted line. Full details are given in Appendix S1 (Supporting information). [Colour figure can be viewed at http://wileyonlinelibrary.com]

- First, consideration must be given to the plastid biology of the group of interest and the availability of a related reference genome. Species with structurally rearranged plastids, such as many holoparasitic plants, will typically be assembled de novo from nonenriched gDNA or isolated plastids, as enrichment strategies relying on PCR primers or baits may not be suitable due to reduced sequence conservation. Similar strategies are often employed for generating a new reference plastome from a given clade, with different strategies such as lower‐coverage sequencing or enrichment for additional individuals (e.g. Curci *et al*. 2015).
- The choice to enrich also depends on the nuclear genome size of the species of interest. Land plant genomes vary 2400‐fold in size, from 61 Mb in the carnivorous plant *Genlisea tuberosa* (Lentibulariaceae) to 149 Gb in *Paris japonica* (Melanthiaceae). The larger the nuclear genome size, the smaller the number of reads in a gDNA sample that will match the plastome and thus the lower plastid sequencing coverage (Fig. 2). As such, enrichment becomes increasingly important for species with large genome sizes, which include many economically important species (e.g. many pines, orchids and wheat).
- Finally, the library preparation approach may simply be dictated by the type of expertise within a research group, with groups with wet‐laboratory technical expertise (or limited access to NGS) more likely to use enrichment, and groups with bioinformatic expertise more likely to directly sequence unenriched gDNA. However, the increasing availability of NGS, and bioinformatics tools for plastid assembly, means gDNA sequencing is likely to become the sample type of choice.

## Library preparation strategies

### Direct sequencing of genomic DNA

A genomic DNA (gDNA) sample contains a mix of nuclear and organellar DNA (plastid and mitochondrion). Thus, in many cases, the plastid can be assembled directly from a gDNA next‐generation sequencing (NGS) library, without prior enrichment or isolation of plastid DNA (Nock *et al*. 2011). In particular, it is becoming popular to perform a ‘genome skim’ (Straub *et al*. 2012), where gDNA is sequenced at low nuclear genome coverage (~0.1–10×), and this often provides sufficient data for complete plastid assembly (Coissac *et al*. 2016). This approach circumvents the need for optimizing species‐specific enrichment protocols (see below) and thus has dramatically streamlined plastome sequencing. This is perhaps the ‘gold standard’ for plastome assembly, often being relatively quick and cheap, and usually leading to high‐quality complete sequence assemblies. While genome skims have proven successful even for degraded herbarium material (Staats *et al*. 2013), special attention may be required during assembly (Box 3).

Box 3 **Strategies for technically challenging plastome assemblies**

Structurally rearranged plastomes of mycoheterotrophs and parasitic plants, as well as degraded herbarium DNA samples, present particular challenges for current plastome assembly workflows. However, new tools are promising for overcoming many of the current limitations.

Herbarium samples present the joint challenge of low levels of recoverable DNA, in conjunction with high sample degradation. Low DNA yields are best overcome by optimizing DNA extraction (Savolainen *et al*. 1995), and the use of low‐input DNA library preparation kits (e.g. NuGEN Ovation Ultralow Library System), or via target enrichment with hybrid baits. Current consensus is that even the most degraded herbarium samples contain DNA potentially suitable for genomic analysis (Staats *et al*. 2013; Bakker *et al*. 2016). DNA degradation may impact the quality of NGS library preparations, because the shearing of poor quality template DNA will result in nonuniform bands. Downstream, assembly of plastomes from herbarium material may be fragmented or incomplete, while a minority may fail entirely (Bakker *et al*. 2016). In practice, it seems that some sample failure is inevitable and may be a limitation that cannot be overcome via new sequence technologies and pipelines. However, even partial plastomes are sufficient for many applications such as phylogenetic reconstruction.

Structurally atypical plastids, such as those of parasitic plants and mycoheterotrophs, often contain rearrangements, pseudogenes and gene deletions. This makes these plastids difficult to assemble using pipelines based on sequence conservation to plastid sequence databases. They may also pose difficulties for de novo plastid assembly pipelines due to low plastid copy number, or unusual GC‐content. As such, assembling a circularized plastome typically required bioinformatic refinement or additional laboratory work (e.g. Naumann *et al*. 2016). Solutions to streamline this process lie both in the generation of sequence data and in improved assembly pipelines. For example, sequence technologies generating reads many Kb in length will greatly facilitate de novo assembly in these groups, resulting in less need to connect scaffolds of unknown order. Improved de novo pipelines using read extension will make it possible to assemble accurate circularized plastome sequences, as has recently been shown with holoparasitic *Cytinus hypocistic* (Roquet *et al*. 2016). Overall it seems technological solutions will improve assembly in groups that have traditionally been a challenge for complete plastome sequencing.

There is a large choice of suitable NGS library types for these gDNA samples, including commonly used PCR‐based libraries (such as Illumina TruSeq, Illumina Nextera and Illumina compatible libraries such as NEB Ultra) and increasingly popular PCR‐free libraries that are less‐error prone but require more input DNA (>1 μg). While library preparation costs vary greatly, many service providers now charge less than $125 per sample. Some of these library preparations can be automated with robot liquid handlers to increase throughput (e.g. Illumina with the Neoprep). A gDNA library will contain a variable amount of plastid data depending on the nuclear genome size and tissue type, with <0.5% plastid reads in some gDNA samples of sugarcane (Hoang *et al*. 2015) to over 20% in milkweeds [Fig. 2, Table S1 (Supporting information), Straub *et al*. 2012]. Thus, the primary concern is designing a multiplex pooling strategy that sequences the desired number of samples with suitable plastid coverage, and choosing bioinformatic analyses that can correctly assign and assemble plastid sequence reads (discussed later).

While there are major benefits to assembling plastids directly from unenriched gDNA extracts, some laboratories may prefer to enrich their samples and focus sequencing effort only on the plastid (Box 2). We next explore these options.

### Enrichment via plastid isolation

Sequencing plastid isolates is an intuitive route to focus sequencing coverage only on the plastid genome. Intact plastids can be isolated from fresh leaves via a sucrose density gradient, using either a home‐made protocol (e.g. Miflin & Beevers 1974) or a proprietary kit (e.g. Sigma Chloroplast Isolation Kit). It is also possible to isolate organellar DNA by high salt precipitation, or by degrading nuclear DNA in a gDNA extraction with DNase I treatment, although these two approaches can give low yields or contamination with mitochondrial DNA (Shi *et al*. 2012). The isolated plastids are typically recovered at a low yield and may require further amplification before sequencing. The main benefit of this approach is that de novo assembly of the enriched DNA sample is simple and will likely lead to a complete assembly even with a small number of sequence reads. This was the case in a chloroplast extraction optimization study by Shi *et al*. (2012), where 5–10 *μ*g of isolated plastid DNA was subject to short‐read sequencing, with 50 Mb of data giving 100× coverage and a complete assembly.

Despite the benefits, there are substantial limitations to plastid isolation approaches, not least the requirement of large quantities of plant tissue which may exceed 5 g of fresh leaves. Further issues are that isolation protocols typically require species‐specific optimization which may hamper large‐scale comparative studies. The sample may also contain nuclear DNA contamination and thus require bioinformatic filtering. As such, the technical challenge associated with retrieving a high‐yield of intact plastids means that genome skimming or other approaches (discussed below) are increasingly popular alternatives for plastid sequencing.

### Enrichment via methylation‐sensitive capture

Plant organelles demonstrate numerous characteristics that distinguish them from the nuclear genome. One rarely exploited feature is that eukaryotic nuclear genomes possess methylated CpG sites, a form of methylation associated with gene expression, while prokaryote‐derived organelles have dramatically lower total methylation (Feng *et al*. 2010). Yigit *et al*. (2014) showed that gDNA could be partitioned into a high‐methylated‐CpG nuclear fraction, and a fraction of low‐methylated‐CpG elements. The methyl‐poor fraction was enriched for plastids by 3.2‐ to 11.2‐fold, depending on the species in question. Subsequent to enrichment, the NGS library for each sample is prepared using standard protocols. Methylation‐sensitive capture is promising as it does not require a priori knowledge of the sequence of interest, and is inexpensive (~$30/sample). However, it requires careful testing before being widely adopted. For example, it is already apparent that this route will not be viable for degraded DNA samples such as herbarium material, where short gDNA fragments may lack a methylated site (here a CpG island), and thus will not be partitioned correctly.

### Enrichment via hybrid bait capture

An alternative way to enrich for plastid DNA is through the use of oligonucleotide probes designed to capture complete plastids. In sequence capture, short probes (‘baits’) are used to isolate complementary sequences from a genomic DNA extract. Post‐capture library pools are then sequenced with NGS. For example, Stull *et al*. (2013) designed a collection of 55 000 baits for eudicots, with each bait intended to capture 120 bp sequences, with a 50‐bp overlap. Their approach worked for enriching a broad range of angiosperm gDNA extracts for plastid DNA. Sequence capture is extremely promising, especially as it would be suitable for a wide‐range of plant material including degraded herbarium samples, and thus deserves further development. In particular, it will be valuable to find less expensive alternatives to the rather expensive commercial enrichment kits (e.g. SureSelect Reagent Kit $1120/16 samples). One example would be MYcroarray, which has been successfully used in a plastid study by Comer *et al*. (2015). In addition to cost, another drawback is the potential to enrich for nuclear‐encoded plastid genes or plastid genes transferred to mitochondria (Box 3). Given the initial expenditure usually associated with this approach, and the subsequent high level of multiplexing required to fill a lane of sequencing, it is best suited to large‐scale analyses of plastomes and is a promising route for whole‐plastid DNA barcoding.

### Enrichment via PCR

PCR is an effective way to enrich a gDNA extract for plastid DNA. The small size (c.150 Kb) and conserved sequence of plastids make it feasible to amplify the complete plastid genome either with short‐range PCR and Sanger Sequencing, or long‐range PCR and NGS. A set of universal primers has been developed to amplify the entire angiosperm plastome in 138 PCRs, with amplicons 0.8–1.5 Kb in length (Dong *et al*. 2013; however, see Prince 2015 for critique of the primers). These amplicons are easy to assemble as they have been designed to overlap by c.100 bp. Short‐range PCR has been successfully used to assemble a wide‐range of representative taxa across the angiosperms. There are also clade‐specific primer sets available for short‐range amplification of plastid DNA (e.g. for monocots, Scarcelli *et al*. 2011). Short‐range PCR represents one of the easiest ways to obtain (near) complete plastids for research laboratories with limited access to NGS or without bioinformatics expertise. However, it does present major limitations. First, it does not scale‐well. Unlike assembly from NGS reads from genomic DNA, which can be highly automated, the Sanger approach requires manual laboratory handling and scoring of sequence chromatograms. Moreover, this approach is only suited to ‘typical’ plastids (see Box 1), and even so the assembly of some regions, such as the boundaries of the inverted repeat, repeat‐rich regions or rapidly evolving genes such as *mat*K and *ycf1*, may require the design of species‐specific primers. As such, short‐range PCR is better suited to applications requiring partial plastids (e.g. population genetic studies such as Whittall *et al*. 2010), rather than complete assemblies (e.g. studies of plastid genome evolution).

The second PCR‐based approach is long‐range PCR and NGS. Yang *et al*. (2014) and Uribe‐Convers *et al*. (2014) have developed suites of universal primers for the long‐range amplification of plastomes in amplicons of 4–23 Kb in length. These large amplicons are then sequenced on an NGS platform. The reduced number of primers relative to the short‐range PCR approach makes this method less time‐consuming in the laboratory, and the longer amplicon size allows all primers to be anchored in low variability regions of the genome. The tagging of different amplicons also allows the multiplexing of many individuals in a single lane of NGS. However, as a PCR‐based approach, it shares limitations outlined above in terms of amplifying known genome regions, and the failure of a single PCR will result in a large gap in the assembled sequence. The large amplicon size also requires high molecular weight DNA, which can be a limitation when working with degraded DNA samples such as herbarium material (e.g. Staats *et al*. 2013). Except in cases where PCR and ligation of barcoded adapters are automated (e.g. Uribe‐Convers *et al*. 2016), long‐range PCR approaches have all the challenges associated with NGS library preparation (expensive and time‐consuming) but without the benefits of direct assembly from gDNA.

## Sequencing strategies

Once a library preparation approach has been chosen, the next choice is picking a sequencing strategy to match. The goal of producing high‐quality complete plastids is increasingly feasible with NGS data. In general, read lengths of current NGS platforms (e.g. 100 bp or longer, paired‐end sequences) have overcome the threshold of repeats in the plastome and thus are sufficient for de novo assemblies (Malé *et al*. 2014). This is a great improvement from early short NGS reads, such as the 36‐bp reads used to assemble the plastome of *Pinus* in up to 183 contigs (Cronn *et al*. 2008). As such, priority should be given to the use of long reads and/or the use of paired‐end data (Straub *et al*. 2012).

**Figure 3:**
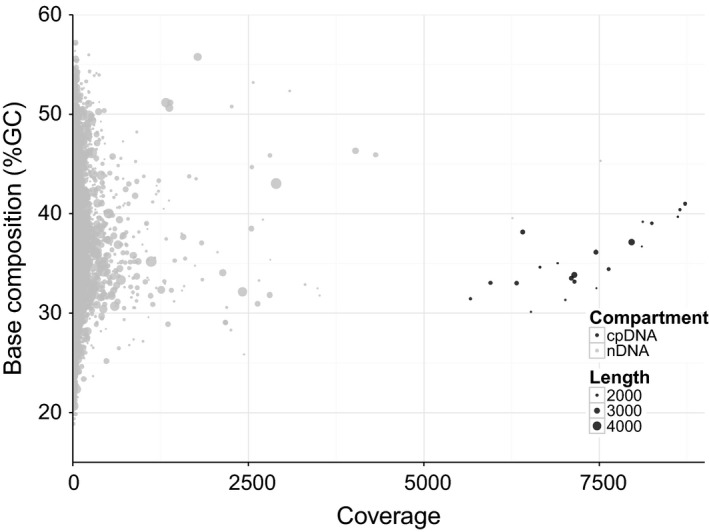
GC by coverage plot of a draft genome assembly. Short reads of the monkey flower *Mimulus guttatus* (population IM, SRR010318) were assembled with SPAdes genome assembler (v3.7.1) and annotated according to matches to the published *Mimulus* plastome (Vallejo‐Marín *et al*. 2016).

When designing a plastid sequencing study, 30× should be considered the minimum planned plastid sequence coverage, with >100× usually desirable. There appears to be no benefit of having very high coverage (over ~200x, A. D. Twyford, Unpublished). As a ballpark figure for sequencing gDNA, 500 Mb of sequence data should be sufficient to assemble the plastid for a typical leaf gDNA extract from a species with a small genome. For example, the ratio of plastid to nuclear genome coverage in gDNA libraries of *Mimulus guttatus* (Phrymaceae, 440 Mb genome size, Fig. 3) is approximately 67:1, which implies that ~3.1% of reads are derived from the plastid. If we sequenced 500 Mb of data, it would result in ~100× coverage of the plastid. Given the small plastid genome size, it is the ideal sample type to run on lower output machines such as the Illumina MiniSeq or MiSeq (Twyford 2016), or other platforms such as Ion Torrent PGM. This would particularly be the case for enriched libraries. For larger numbers of genome skims, it would be more cost‐effective to use high‐output sequencers such as the Illumina HiSeq 4000 (750 Gb/run); see http://www.molecularecologist.com/next-gen-fieldguide-2016 for comparison of sequencing platforms. As sequencing output increases, the potential number of plastids that can be pooled in a single sequencing run is very large (many 100s). This is made possible by the growing number of available adapters (e.g. commercial 384‐plex adapter sets) and dual‐indexing strategies (Sickel *et al*. 2015).

Long‐read third‐generation sequencing is extremely promising for the assembly of small genomes such as plastids. For example, Pacific Biosciences long‐read sequencers typically generates reads >15 Kb, with the longest reads up to 60 Kb in length. These long reads, in conjunction with lack of bias in AT‐rich regions, make it ideal for plastid assembly. The current high costs on a per‐read or per‐Mb basis means isolation of plastid DNA or enrichment is commonly done prior to sequencing (e.g. >100× coverage, Wu *et al*. 2014; Chen *et al*. 2015), although gDNA has also been sequenced (c.2000× plastid coverage, Stadermann *et al*. 2015). These long sequence reads are becoming increasingly cost‐effective as new platforms are released, such as the PacBio Sequel.

## Assembly

Plastid sequence reads can be assembled to a reference genome or de novo. Reference‐guided assembly is most well suited to studies of related taxa where a reference genome exists. De novo assembly is preferable across phylogenetically divergent groups, species without available reference sequences, or groups with structural rearrangements or major gene loss or genome expansion (Fig. 1b,c, Box 3).

A common first stage in many de novo plastid assembly approaches is to separate plastid reads from nuclear and mitochondrial reads. Filtering before assembly can be an important way to reduce the complexity of a library, which greatly facilitates de novo assembly. Moreover, because the expected coverage of plastid reads is so much higher, assembling before filtering can lead to problems with error correction and de novo assembly algorithms that expect even coverage. Only in instances where researchers have a priori information that plastomes could have atypical gene content or copy number would it be necessary to conduct assembly before filtering plastid‐derived sequences, such as in the highly rearranged plastome of the parasitic plant *Hydnora* (Naumann *et al*. 2016).

There are two nonmutually exclusive approaches for isolating plastid reads; first, they can be separated based on similarity to known plastid sequence. For example, reads can be matched to a database of plastid sequences using blast or aligned to a related plastid using a short‐read aligner like Bowtie 2 (Langmead & Salzberg 2012). This may lead to some gaps in regions divergent from the reference sequence or database. Stringent read filtering by sequence similarity is not advised in lineages with atypical plastome structure or from lineages where no close reference sequence exists, as this can lead to incomplete assemblies. This filtering strategy also has the downside that it may incorrectly remove mitochondrial DNA that has been transferred to the plastid genome (Iorizzo *et al*. 2012; Straub *et al*. 2013; Ma *et al*. 2015; Wysocki *et al*. 2015). Additional plastid reads can be recovered using read extension approaches such as the GetOrganelle script (https://github.com/Kinggerm/GetOrganelle). Here, the first set of reads matching reference plastid(s) are used as seeds for successive rounds of extension, where additional reads that overlap the seeds are incorporated into the pool of plastid reads.

A second approach to recover plastid reads in gDNA is from distinct properties of the plastid, rather than similarity to known sequences. In particular, plastid reads are usually present with many‐fold higher coverage than nuclear DNA (though see Box 3). For example, a genome skimming study in Jerusalem artichoke (*Helianthus tuberosus*) found plastid DNA had over 1400 times higher coverage than the 9.4 Gbp nuclear genome (plastid DNA = 355×, nDNA = 0.25×), and an approximately 20‐fold greater coverage than the mitochondrial genome (Bock *et al*. 2014). Second, most land plant plastids have a distinct GC‐content to the nuclear genome (plastid DNA ~37%, Civáň *et al*. 2014; nDNA ~41% Li & Du 2014), although this distinction is not always clear (Smith *et al*. 2011; Šmarda *et al*. 2012). Taking these properties together, plots of GC‐content against read depth can be effective for distinguishing plastid reads (Fig. 3). This approach can be combined with best‐matching sequences in annotated databases to provide an effective filtering strategy (Kumar *et al*. 2013). An advantage of using coverage and GC‐content rather than similarity is that it may remove potentially problematic regions where plastid genes have been translocated to the nuclear genome and share sequence properties with nuclear rather than plastid DNA (Oliver *et al*. 1990). Similarly, by breaking raw reads into pieces of length k (so‐called kmers), we can count the frequency with which each kmer occurs using software such as BFcounter (Melsted & Pritchard 2011). The resulting count distribution, known as the kmer frequency, can be used to extract reads from high‐copy DNA such as the plastome.

Suitable de novo assemblers for these filtered plastid reads include ABySS, CLC Genomic Workbench, Edena, Euler‐sr, Geneious de novo, MIRA, Newbler, SOAPdenovo, SPAdes, SSAKE or Velvet (reviewed in Ekblom & Wolf 2014). In many cases, assembly performance and run‐time may be improved by down sampling the number of reads. A de novo assembly with a large kmer value, typically with minimal optimization of assembly parameters, will often yield good results assembling the plastid into a small number of large contigs. Further refinements are required to join scaffolds such as those broken by the inverted repeats (discussed below).

Instead of filtering nonplastid reads prior to assembly, there are a growing number of programs (mitobim, org.asm, fast‐plast) that merge filtering with assembly. The approach is to use known plastid (or mitochondrial) sequence as seeds to identify or ‘bait’ plastid reads and approximate coverage. From these seeds, assembly proceeds by finding reads that overlap the reads already incorporated. The organelle asembler (org.asm, http://pythonhosted.org/ORG.asm) uses baiting followed by cycles of stack filling, extension, cleaning and gap filling to assemble circular plastomes. It is reported to return 70% of plastids as complete (Coissac *et al*. 2016). The assembly software fast‐plast (https://github.com/mrmckain) is similar in its use of seed‐based baiting, but also uses a conventional assembler and is designed to correctly orientate the inverted repeat. These assemblers are the most direct means to produce circularized assemblies and can be highly automated for large sample sizes. However, the lack of published comparisons with other assemblers means careful examination should be given to the assembly quality particularly in repetitive regions. It is also unclear how well they perform in structurally atypical plastids such as those found in parasitic plants or whether it always accurately assembles the full plastome including both inverted repeats (A. D. Twyford & R. W. Ness, Unpublished).

The most common outcome of de novo plastid assembly is a small number of long contigs with breaks corresponding to the large single copy (LSC), small single copy (SSC) and inverted‐repeat (IR) regions. This is because many assemblers struggle to cope with the pair of near identical IRs, and as such collapse both IRs and display double the read depth for this region. These contigs can subsequently be stitched together, bearing in mind that plastids exist in two different states within a cell with alternate SSC orientation (Walker *et al*. 2015). Care should be given to check reads bridging the IR‐boundary give an accurate sequence assembly. For studies where precise IR boundaries are important, any remaining uncertainty can be examined using PCR primers that span IR boundaries. This plastid finishing step is however increasingly unnecessary with methods using read extension or approaches using long sequence reads.

While plastome assembly can be a routine and easy task from DNA extracted from fresh tissue of autotrophic land plants, this is not always the case. One relatively common yet often unexpected issue is intracellular gene transfer (Iorizzo *et al*. 2012; Straub *et al*. 2013; Ma *et al*. 2015; : Wysocki *et al*. 2015). It is now apparent that plastids can exchange DNA with the nucleus and mitochondria. Foreign DNA in plastids (and plastid DNA in the mitochondria and nucleus) can often be distinguished from unique properties of the plastome, described above, such as copy number. This must be accounted for to complete a plastome sequence.

## Conclusions

Plastome sequencing is at an exciting turning point. Large‐scale NGS library preparation, increasing read lengths and sequencing throughput, and automated assembly pipelines, make the prospect of plastid sequences for all lineages of land plants and algae a real possibility. These plastid sequences can increasingly be harnessed to their full potential with improved downstream processing including automatic annotation (Huang & Cronk 2015) and many integrated pipelines suited to large data sets (such as The Plastome Database, http://verdant.iplantcollaborative.org/plastidDB/). These data have great potential for increasing our understanding of plant biology and genome evolution and will set the context for future exploration of plastid gene expression (Sanitá Lima *et al*. 2016), as well as complementary investigations of the nuclear genome.

A.D.T. and R.W.N. wrote the manuscript and prepared the figures.

## Supporting information


**Appendix S1** Proportion of plastid reads in gDNA sequence libraries of species with different genome sizes.
