# Supplementary material for: Strategies for complete plastid genome sequencing
Source: Mol Ecol Resour. 2016 Nov 28;17(5):858–68. doi: 10.1111/1755-0998.12626 (PMC6849563; doi:10.1111/1755-0998.12626)
Supplement: Supplementary file 1 — Appendix S1 Proportion of plastid reads in gDNA sequence libraries of species with different genome sizes. [file MEN-17-858-s001.docx]

**Appendix S1. Proportion of plastid reads in gDNA sequence libraries of species with different genome sizes.**

Twenty seven plant species with archived short sequence reads from gDNA libraries, and with known genome sizes, were selected to represent the major green plant lineages and the full range of plant genome sizes. Genome size estimates were downloaded from the Kew Plant DNA C-value Database (Bennett & Leitch, 2012, <http://data.kew.org/cvalues>) except where noted, and values were converted to megabases with the conversion 1 pg = 978 Mb. One hundred thousand SRA reads were downloaded per taxa and mapped to a reference database of 109 plastid genomes (Cui *et al.* 2006) using the map to reference option in Geneious v9.1.5.

| Species | Sequence Read Archive accession number | Genome size (Mb) | Proportion plastid reads | Reference |
| --- | --- | --- | --- | --- |
| *Galdieria phlegrea* | SRR953992 | 14 | 0.06921 | Qiu *et al.* (2013) |
| *Cyanidioschyzon merolae* | SRR1002342 | 17 | 0.01303 | Matsuzaki *et al.* (2004) |
| *Genlisea nigrocaulis* | ERR412884 | 86 | 0.10389 | Tran et al. (2015) |
| *Utricularia gibba* | SRR768511 | 88 | 0.02144 | Kew database |
| *Arabidopsis thaliana* | ERR1404860 | 156 | 0.16415 | Kew database |
| *Capsella rubella* | ERR636174 | 215 | 0.27211 | Kew database |
| *Boechera stricta* | SRR1801050 | 235 | 0.10855 | Kew database |
| *Prunus armeniaca* | SRR2153164 | 293 | 0.03454 | Kew database |
| *Arabidopsis arenosa* | SRR2082785 | 196 | 0.08743 | Kew database |
| *Asclepias syriaca* | SRR098429 | 411 | 0.37283 | Kew database |
| *Mimulus guttatus* | SRR052272 | 362 | 0.02529 | Kew database |
| *Phaseolus vulgaris* | SRR071416 | 587 | 0.04682 | Kew database |
| *Sorghum bicolor* | SRR3204498 | 734 | 0.01545 | Kew database |
| *Ipomoea trifida* | DRR023898 | 831 | 0.04029 | Kew database |
| *Plantago lagopus* | ERR1124257 | 1223 | 0.02089 | Kew database |
| *Senecio vulgaris* | SRR2155042 | 1545 | 0.05827 | Kew database |
| *Tragopogon dubius* | SRR585565 | 2875 | 0.06678 | Kew database |
| *Helianthus annuus* | SRR2919291 | 2377 | 0.02807 | Kew database |
| *Lolium perenne* | ERR1333497 | 2699 | 0.01339 | Kew database |
| *Capsicum annuum* | SRR2752016 | 3090 | 0.0164 | Kew database |
| *Welwitschia mirabilis* | ERR845262 | 7042 | 0.05634 | Kew database |
| *Lathyrus latifolius* | ERR413120 | 10641 | 0.01899 | Kew database |
| *Vicia faba* | SRR3656962 | 13037 | 0.06901 | Kew database |
| *Allium cepa* | SRR1265913 | 16382 | 0.02065 | Kew database |
| *Picea abies* | ERR268242 | 19570 | 0.00271 | Kew database |
| *Pinus taeda* | SRR1054298 | 21614 | 0.00635 | Kew database |
| *Fritillaria imperialis* | ERR845263 | 42005 | 0.01346 | Kew database |

**References**

Cui L, Veeraraghavan N, Richter A*, et al.* (2006) ChloroplastDB: the Chloroplast Genome Database. *Nucleic Acids Research* **34**, D692-D696.

Qiu H, Price DC, Weber APM*, et al.* Adaptation through horizontal gene transfer in the cryptoendolithic red alga *Galdieria phlegrea*. *Current Biology* **23**, R865-R866.

Matsuzaki M, Misumi O, Shin-i T*, et al.* (2004) Genome sequence of the ultrasmall unicellular red alga *Cyanidioschyzon merolae* 10D. *Nature* **428**, 653-657.

Tran TD, Cao HX, Jovtchev G*, et al.* (2015) Chromatin organization and cytological features of carnivorous *Genlisea* species with large genome size differences. *Frontiers in Plant Science* **6**, 613.
